# Collapsing glomerulopathy in sickle cell disease: a case report

**DOI:** 10.1186/1752-1947-5-71

**Published:** 2011-02-21

**Authors:** Ganga B Ramidi, Mohan K Kurukumbi, Peter L Sealy

**Affiliations:** 1 Department of Internal Medicine, Howard University Hospital, 2041 Georgia Avenue, Washington, DC 20060, USA; 2 Department of Neurology, Howard University Hospital, 2041 Georgia Avenue, Washington, DC 20060, USA

## Abstract

**Introduction:**

Sickle cell disease has been associated with many renal structural and functional abnormalities. Collapsing glomerulopathy or the collapsing variant of focal segmental glomerulosclerosis is a rare clinicopathologic entity in patients with sickle cell disease that requires timely diagnosis and aggressive management.

**Case presentation:**

In this case report we describe a 21-year-old African-American woman with a medical history of significant sickle cell disease and asthma. She was admitted for pain, decreased urine output, bilateral leg swelling and reported weight gain. During her period of hospitalisation she developed acute renal failure requiring dialysis. Further investigation revealed the collapsing variant of focal segmental glomerulosclerosis.

**Conclusions:**

Although focal segmental glomerulosclerosis is a common feature of sickle cell nephropathy, the collapsing variant of focal segmental glomerulosclerosis or collapsing glomerulopathy has been rarely documented. Even when other risk factors are controlled, collapsing glomerulopathy has a very poor prognosis. This is a rare case of a patient with massive proteinuria presenting as acute renal failure with a very poor response to corticosteroids and a much faster rate of progression to end-stage renal disease.

## Introduction

The renal features of sickle cell disease (SCD) include hematuria, proteinuria, tubular disturbances and chronic kidney disease [1]. Proteinuria is more commonly encountered in patients with homozygous (hemoglobin SS) SCD than in other hemoglobinopathies [2]. Proteinuria occurs in 20% to 30% of patients with SCD, although a higher incidence has also been reported. The morphologic lesions most frequently identified in SCD are focal segmental glomerulosclerosis (FSGS) and membranoproliferative glomerulonephritis (MPGN)-like disease without immune complex deposits. FSGS causes about 10% to 15% of all cases of nephrotic syndrome in SCD. However, the collapsing variant of FSGS or collapsing glomerulopathy (CG) has rarely been documented in SCD. In the literature, there are only a few reports of CG in patients with SCD [3]. In this report, we present another non-human immunodeficiency virus (non-HIV) patient with SCD who had a rare association of CG and SCD.

## Case presentation

Our patient was a 21-year-old African-American woman with SCD-SS and asthma. She presented with shortness of breath, generalized body pain and nonproductive cough along with fever of two weeks' duration. She was admitted for pain and presumed pneumonia. She had sickle cell crisis only once yearly and had not required any blood transfusions or hydroxyurea treatment in the past.

On review of her systems, some positive pertinent findings included decreased urine output, bilateral leg swelling and weight gain. She had no known drug allergy or illicit drug use. Her family history was significant for the sickle cell trait in her parents. There was no history of significant kidney disease or recent travel.

On examination, she was found to be obese (body mass index > 30 kg/m^2^) and febrile (temperature 102°F) with blood pressure of 105/48 mmHg, heart rate of 90 beats/min, respiratory rate of 18/min and oxygen saturation of 100% on room air. Pertinent physical examination findings included pallor, periorbital edema, extremities with 3+ pedal edema bilaterally and no skin rash. All other systems were normal.

Initial laboratory work showed that she had a white blood cell count of 31 × 10^9^/L, neutrophils 90%, bands 9%, hemoglobin 8.9 mmol/L, hematocrit 24.8%, a platelet count of 446 × 10^9^/L, blood urea nitrogen (BUN) 8.57 mmol/L and creatinine 79.95 μmol/L. Her serum electrolytes were normal, and her liver function tests were normal except for total protein 0.05 g/L and serum albumin 0.02 g/L. Urine analysis showed 3+ proteinuria (10 g/d, 70% albuminuria) as well as glucosuria with specific gravity of 1.023. The patient's blood, urine and sputum cultures were negative. Her chest X-ray was normal.

Our patient was started on empiric antibiotic treatment with ceftriaxone and azithromycin for presumed pneumonia. However, on the second day of admission, her creatinine level increased from 79.95 μmol/L to 300.56 μmol/L and she developed anasarca. The nephrology department was consulted, and immediate dialysis was initiated for acute renal failure (ARF).

On the basis of the patient's gender, age, hypoalbuminemia, proteinuria (10 g/day; 70% albumin) and microscopic hematuria, lupus nephritis was suspected. Serological tests for anti-neutrophil antibody (ANA), rheumatoid factor (RF), anticardiolipin antibodies and lupus anticoagulant were negative. Ultrasound of the kidneys showed echogenic kidneys compatible with medical renal disease. The right and left kidneys measured 10.9 cm in sagittal dimensions. A percutaneous renal biopsy was performed, and a total of 30 glomeruli were examined. Light microscopy revealed a profoundly altered glomerular filtration barrier in more than 20 glomeruli. Hyperplastic and hypertrophic podocytes or pseudocrescents were seen in most glomeruli. Electron microscopy revealed focal segmental collapse of glomerular capillaries and basement membrane with extensive foot process effacement in approximately 10 glomeruli. Immunofluorescence staining was negative for immunoglobulin G (IgG), IgA, IgM, C1q, C3, fibrin, albumin and κ and λ chains. A diagnosis of CG was considered and lupus was excluded. Extensive searches for the main causes of CG were negative. Parvovirus B19 (PVB19) serology found IgG but no IgM, whereas PVB19 DNA polymerase chain reaction was negative in peripheral blood.

The following serologies were negative: HIV, cytomegalovirus (CMV), hepatitis C virus, hepatitis B virus, *Treponema pallidum *hemagglutination-Venereal Disease Research Laboratory (VDRL), Lyme, borreliosis, *Leishmania *and *Loa loa*. The patient's blood HIV DNA polymerase chain reaction was negative at the time of diagnosis and after six months. She was not a drug user and had never received pamidronate, lithium or interferon-α.

Our patient was treated with intravenous prednisolone (1 mg/kg/day) and was later switched to oral prednisone (40 mg/day). Prednisone was maintained at adequate doses for eight weeks and later changed to tapering doses. Our patient was treated with hemodialysis on alternate days for 21 days, followed by maintenance hemodialysis for two months. She showed marked clinical improvement along with improved renal function. However, she had a relapse of disease after two months. She was again presented with ARF and eventually progressed into end-stage renal disease (ESRD), requiring hemodialysis. We followed the patient for one year, but she was lost to follow-up.

## Discussion

**Figure 1:**
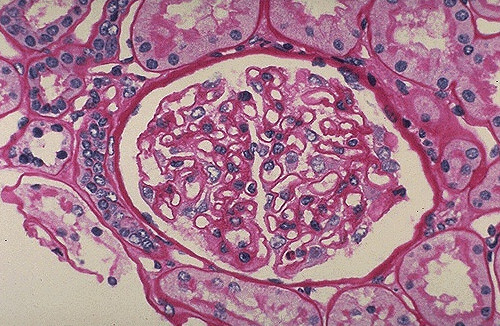
**Normal glomerulus**. This normal glomerulus is stained with periodic acid-Schiff (PAS) stain to highlight the basement membranes of glomerular capillary loops and tubular epithelium. The capillary loops of this normal glomerulus are well defined and thin.

Sickle cell nephropathy (SCN) is an important cause of mortality and morbidity in patients with SCD. Sickle cell anemia (SCA) and the related hemoglobinopathies are associated with a large spectrum of renal abnormalities (Figure 1) [4]. SCN is a complex entity characterized by decreased medullary blood flow, hyposthenuria, hematuria, ischemia, microinfarct and papillary necrosis [5,6]. Patients with SCD have impaired urinary concentrating ability, defects in urinary acidification and potassium excretion and supranormal proximal tubular function. Young patients with SCD have supranormal renal hemodynamics with elevations in both effective renal plasma flow (ERPF) and glomerular filtration rate (GFR). These parameters decrease with age as well as with administration of prostaglandin inhibitors.

Proteinuria, a common finding in adults with SCD, may progress to the nephrotic syndrome. Proteinuria, hypertension and increasing anemia predict ESRD [7]. The incidence of renal failure in patients with nephritic syndrome and SCD ranges from 5% to 18%, and the severity of renal insufficiency has been found to be age related. Individuals with SCD-SS have been known to develop nephrotic syndrome from poststreptococcal glomerulonephritis (PSGN), membranoproliferative glomerulopathy (MPGN) and minimal change disease (MCD). Most importantly, glomerular injury specific to the SCD-SS state may also result in nephrotic syndrome.

FSGS causes about 10% to 15% of all cases of nephrotic syndrome in patients with SCD-SS of SCN. FSGS is a disease with diverse histological patterns and etiologic associations, occurring in two types: primary or idiopathic and secondary forms. Variants of primary FSGS and incidence are as follows: collapsing form (11%), cellular variant (3%), perihilar (26%), tip lesions (17%) and FSGS not otherwise specified (42%) [8-11].

**Figure 2:**
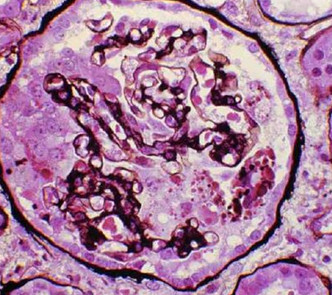
**Collapsing variant of focal segmental glomerulosclerosis (FSGS)**. The collapsing form of FSGS is a histologic variant which is characterized by mesangial hypercellularity and resultant collapse of the glomerular capillaries.

CG (Figure 2) is a morphologic variant of FSGS characterized by segmental and global collapse of the glomerular capillaries, marked hypertrophy and hyperplasia of podocytes and severe tubulointerstitial disease [12,13].

The first description of the disease appeared in 1978 and was named "malignant focal segmentalglomerulosclerosis" because of rapidly progressive nephrotic syndrome [7,14]. In the early 1980s, during the HIV pandemic, "HIV-associated nephropathy" was the common term to identify the injury. In 1986, Weiss *et al*. [14] described a similar renal lesion in HIV-negative patients with severe proteinuria and rapid progression to renal failure, and the term "collapsing glomerulopathy" was used for the first time to indicate this new clinical-pathologic entity.

Numerous hypotheses for the pathogenesis of CG have been generated, but no specific common trigger for epithelial cell proliferation has emerged. It is postulated that conditions of poor oxygenation will lead to glomerular enlargement and predisposition to CG. Its pathogenesis was thought to involve visceral epithelial cell injury leading to podocyte dedifferentiation and detachment from the glomerular basement membrane. The prevalence of the disease in blacks suggests a genetic susceptibility [15,16]. Identification of mutations in the chromosome encoding for CoQ2 in a European family and prenyltransferase-like mitochondrial protein in the *kd/kd *mouse further corroborates the genetic susceptibility [16,17].

Most cases of CG have been associated with HIV infections. Other secondary causes of CG include pamidronate therapy, parvovirus B19 infection, hepatitis C virus (HCV), interferon-α (IFN-α) treatment for hepatitis, CMV infection, human T-cell lymphotrophic virus and immune deficiencies. CG also may recur after renal transplantation or present *de novo*, often leading to loss of the allograft.

Clinically, CG is characterized by black racial predominance, high levels of nephrotic range proteinuria, rapidly progressive renal failure, marked parenchymal injury and poor response to present therapeutic regimens.

CG in SCD, although uncommon, is increasingly recognized, predominantly in African-Americans with SCD. In the literature, only a few cases of CG in SCN have been reported. Bhathena and Sondheimer [3] reported six cases of homozygous sickle cell patients who had renal transplant with CG and rapid progression to chronic renal failure. This case report presents a unique case of CG in SCN in an African-American non-HIV native kidney patient.

An effective therapeutic regimen for CG in SCD has not been clearly defined, and no evidence-based therapy exists for CG [18,19]. The current recommendations for CG treatment are based on anecdote and expert opinion. The leading recommendations for the treatment of CG in non-HIV-infected patients suggest drug regimens that are used to treat FSGS. It is important to note that the mechanism by which the podocytes are injured is different in FSGS and CG. In FSGS, segmental solidification (sclerosis) of the tuft with adhesion to Bowman's capsule occurs. However, in CG, there is a collapse of glomeruli and pseudocrescent formation. CG is characterized by proliferation of podocytes, whereas podocytopenia is implicated in the pathogenesis of FSGS. Thus, it is not surprising that CG is resistant to standard therapies used for FSGS and should be recognized as a distinct entity.

## Conclusions

Collapsing FSGS or CG has been rarely documented in SCD. CG has a very poor prognosis. We present a rare case of massive proteinuria along with a much faster rate of progression to ESRD. SCN with CG is a rare clinicopathologic entity which requires further research.

## Abbreviations

ANA: antineutrophil antibody; ARF: acute renal failure; CG: collapsing glomerulopathy; ERPF: effective renal plasma flow; ESRD: end-stage renal disease; GFR: glomerular filtration rate; FSGS: focal segmental glomerulosclerosis; MPGN: membranoproliferative glomerulopathy; MCD: minimal change disease; PSGN: poststreptococcal glomerulonephritis; RF: rheumatoid factor; SCA: sickle cell anemia; SCD: sickle cell disease; SCN: sickle cell nephropathy; VDRL: Venereal Disease Research Laboratory.

## Competing interests

The authors declare that they have no competing interests.

## Consent

Written informed consent was obtained from the patient for publication of this case report and any accompanying images. A copy of the written consent is available for review by the journal's Editor-in-Chief upon request.

## Authors' contributions

GBR collected the patient data and was involved in patient care and in drafting the manuscript. PLS provided general support. MKK provided technical help and writing assistance. All authors read and approved the final manuscript.
